# Correction to: Treatments, resource utilization, and outcomes of COVID-19 patients presenting to emergency departments across pandemic waves: an observational study by the Canadian COVID-19 Emergency Department Rapid Response Network (CCEDRRN)

**DOI:** 10.1007/s43678-022-00314-z

**Published:** 2022-04-22

**Authors:** Corinne M. Hohl, Rhonda J. Rosychuk, Jeffrey P. Hau, Jake Hayward, Megan Landes, Justin W. Yan, Daniel K. Ting, Michelle Welsford, Patrick M. Archambault, Eric Mercier, Kavish Chandra, Philip Davis, Samuel Vaillancourt, Murdoch Leeies, Serena Small, Laurie J. Morrison, the Canadian COVID-19 Rapid Response Network (CCEDRRN) investigators for the Network of Canadian Emergency Researchers, for the Canadian Critical Care Trials Group

**Affiliations:** 1 Department of Emergency Medicine, University of British Columbia, Vancouver, BC Canada; 2 Centre for Clinical Epidemiology and Evaluation, Vancouver Coastal Health Research Institute, Vancouver, BC Canada; 3 Department of Pediatrics, University of Alberta, Edmonton, AB Canada; 4 Department of Emergency Medicine, University of Alberta, Edmonton, AB Canada; 5 Division of Emergency Medicine, University of Toronto, Toronto, ON Canada; 6 University Health Network, Toronto, ON Canada; 7 Division of Emergency Medicine, London Health Sciences Centre, London, ON Canada; 8 Schulich School of Medicine and Dentistry, Western University, London, ON Canada; 9 Division of Emergency Medicine, McMaster University, Hamilton, ON Canada; 10 Hamilton Health Sciences, Hamilton, ON Canada; 11 Department of Family Medicine and Emergency Medicine, Université Laval, Quebec, QC Canada; 12 Centre de recherche du Centre intégré de santé et de services sociaux de Chaudière-Appalaches, Levis, QC Canada; 13 Centre de Recherche, CHU de Québec, Université Laval, Quebec, QC Canada; 14 VITAM (Centre de recherche en santé durable), Quebec, QC, Canada; 15 Department of Emergency Medicine, Dalhousie Medicine New Brunswick, Saint John, NB Canada; 16 Department of Emergency Medicine, Saint John Regional Hospital, Saint John, NB Canada; 17 Department of Emergency Medicine, University of Saskatchewan, Saskatoon, SK Canada; 18 Department of Emergency Medicine, St Michael’s Hospital, Unity Health Toronto, Toronto, ON Canada; 19 Department of Emergency Medicine, University of Manitoba, Winnipeg, MB Canada; 20 Section of Critical Care Medicine, University of Manitoba, Winnipeg, MB Canada

## Correction to: Canadian Journal of Emergency Medicine 10.1007/s43678-022-00275-3

In this article, the name of the Canadian COVID-19 Rapid Response Network (CCEDRRN) investigators for the Network of Canadian Emergency Researchers, for the Canadian Critical Care Trials Group was incorrect in the author line. The original article has been corrected.

